# A Multidisciplinary Integrated Care Program for Complex Chronic Patients: A Real-World Evaluation of Healthcare Utilization and Provider Costs

**DOI:** 10.3390/healthcare14162542

**Published:** 2026-08-14

**Authors:** Jorge Martins, António Oliveira, Inês Chora, Fernando Friões

**Affiliations:** 1 Internal Medicine Department, ULS Matosinhos, Senhora da Hora, 4464-513 Matosinhos, Portugal; antonio.oliveira@ulsm.min-saude.pt (A.O.); ines.chora@ulsm.min-saude.pt (I.C.); 2 Department of Medicine, Faculty of Medicine, University of Porto, 4200-319 Porto, Portugal; fbfrioes@gmail.com; 3 Internal Medicine Department, ULS São João E.P.E., 4200-319 Porto, Portugal; 4 Cardiovascular Research and Development Center (UnIC@RISE), Faculty of Medicine, University of Porto, 4200-319 Porto, Portugal

**Keywords:** integrated care, complex chronic patients, multimorbidity, case management, healthcare utilization, healthcare costs, real-world evidence, coordinated care

## Abstract

**Background:** Complex chronic patients (CCPs) frequently experience fragmented care and high healthcare utilization. Integrated multidisciplinary care may improve care coordination and reduce reliance on acute healthcare services, although evidence regarding its impact on healthcare utilization and costs remains inconsistent. This study evaluated the real-world impact of a multidisciplinary integrated care program on healthcare utilization and provider costs among CCPs. **Methods:** This retrospective before-and-after study included CCPs enrolled in a multidisciplinary integrated care program between 2016 and 2023. Healthcare utilization and provider costs from the provider perspective were compared for up to two years before and after enrollment. **Results:** The study included 473 patients with a median age of 82 years, 56.7% of whom were women. Significant reductions in acute and ambulatory healthcare utilization and overall provider costs were observed following enrollment and were sustained over two years (*p* < 0.001). Exploratory economic analyses showed that lower provider costs occurred alongside reductions in acute healthcare utilization. **Conclusions:** A multidisciplinary integrated care program for CCPs was associated with sustained reductions in healthcare utilization and provider costs over two years. These findings support the potential value of integrated multidisciplinary care for CCPs.

## 1. Introduction

The prevalence of multimorbidity, defined as the coexistence of two or more chronic conditions in the same individual, was estimated at 37.9% in Europe in 2022 [1] and is expected to continue increasing, largely driven by rising life expectancy and social and medical advances, particularly the ability to transform rapidly fatal diseases into chronic conditions. For example, in England, the prevalence of multimorbidity is projected to increase from 54.0% in 2015 to 64.4% in 2025 and 67.8% by 2035 [2]. This has led to a growing population of patients with multimorbidity, polypharmacy, and high healthcare utilization rates. This population, commonly referred to as complex chronic patients (CCPs), represents a major challenge for healthcare systems, which have traditionally been organized around acute and single-disease care models rather than the complex needs of patients requiring personalized, longitudinal, and coordinated care. Patients with multimorbidity frequently experience fragmented healthcare trajectories characterized by poor coordination between providers, duplication of interventions, inconsistent therapeutic plans, and reactive reliance on acute care services. Furthermore, the substantial healthcare costs associated with this population constitute an additional concern, partly driven by fragmented care pathways that contribute to inefficiencies in healthcare delivery and resource utilization. The cumulative burden of morbidity is a key issue for CCPs [3], contributing to the progressive deterioration of the quality of life. Older patients with multimorbidity and frailty syndrome represent a progressively larger proportion of hospital admissions [4]. Multimorbidity alone is associated with a high utilization of healthcare services, including an increased number of primary care consultations, emergency department visits, and hospital admissions [5].

Integrated care models have emerged as a response to these challenges, aiming to improve coordination across professionals, organizations, and care settings [6] while promoting continuity, person-centered care, and more efficient resource utilization. These approaches frequently combine clinical, organizational, and professional integration strategies to address the multidimensional needs of patients with multimorbidity. Recent integrated care frameworks, such as the IFIC’s Nine Pillars of Integrated Care [7], further emphasize the importance of person-centered care, continuity, multidisciplinary collaboration, community partnerships, and system-wide coordination as key enablers of effective integrated care implementation.

CCP management has focused on case management, follow-up by multidisciplinary teams, and care coordination. Numerous examples of such teams have been described, involving a wide range of healthcare professionals, such as nurses [8], physiotherapists, pharmacists, and social workers, as well as different combinations of chronic conditions and varying degrees of professional participation. This heterogeneity makes it difficult to compare studies in terms of associated outcomes [9] and costs. In a systematic review by Ann-Kathrin Klaehn et al. [10], which included 29 studies on the cost-effectiveness of case management, the intervention was less effective and more costly than the control group in three studies; in two studies, the intervention was less effective and less costly; in six studies, it was more effective and less costly; and in 17 studies, the intervention was more effective but also more costly. Similarly, another review conducted in Germany [11] reported mixed results without favoring any specific intervention when comparing case management with usual care. Further obstacles to drawing conclusions include the specific characteristics of each national health system in terms of cost calculations and whether healthcare management is public or private.

Implementing multidisciplinary integrated care programs requires additional organizational resources and changes in healthcare delivery. Their value therefore depends not only on clinical outcomes but also on their ability to improve coordination of care, optimize healthcare utilization and support more efficient use of healthcare resources. A multidisciplinary team performing personalized care for CCPs represents an additional cost to the health system, requiring a meaningful impact on future resource utilization or an improvement in quality of life to be considered cost-effective. Evaluating the impact of integrated care programs on healthcare utilization and provider costs is therefore essential to inform service planning and health policy. The economic evaluation of case management programs may support a more appropriate selection of patients to be enrolled in these programs, but it does not guarantee a reduction in healthcare costs [12]. The involvement of additional healthcare professionals, particularly physicians, in the follow-up of patients with complex chronic conditions requires careful consideration to avoid the risk of increased chronic medication prescription, a higher volume of diagnostic tests, and a greater number of hospital outpatient visits associated with regular follow-up. The selection of an internal medicine physician or geriatrician may mitigate this risk [13].

This study aimed to evaluate the real-world impact of a multidisciplinary integrated care program for CCPs on healthcare utilization and provider costs [14] within an integrated Local Health Unit. A secondary objective was to assess the association between changes in acute healthcare utilization and healthcare costs.

## 2. Materials and Methods

### 2.1. Study Design and Setting

This was a retrospective longitudinal observational study using a before-and-after design to evaluate the implementation of a multidisciplinary integrated care program for CCPs within an integrated Local Health Unit. The study was designed as a real-world service evaluation aimed at assessing changes in healthcare utilization and provider costs following program implementation rather than estimating the causal effect of the intervention. CCPs enrolled in a case management program between November 2016 and April 2023 were included. Costs from the perspective of the healthcare provider in the two years before the program were compared with those incurred in the two years following enrollment. Direct and indirect costs related to primary care consultations, hospital outpatient visits, emergency department visits, inpatient admissions, and day hospital sessions were considered.

### 2.2. Participants

Patients were classified as CCPs if they met at least three of the following criteria:

1. Age ≥ 75 years;
2. Five or more emergency department visits within the past 365 days;
3. Three or more hospitalizations within the past 365 days;
4. Chronic use of six or more medications;
5. Three or more of the following seven chronic conditions: diabetes mellitus, active cancer, cerebrovascular disease, heart failure, chronic obstructive pulmonary disease/chronic respiratory failure, chronic kidney disease, or chronic liver disease.

Patients were consecutively enrolled after referral to the program according to routine clinical practice. Enrollment into the Complex Chronic Patients Support Service depended on referral by the attending physician from hospital or primary care services, provided that the predefined eligibility criteria were fulfilled.

### 2.3. Integrated Care Intervention

The CCP follow-up program operates as a functional unit, designated as the Complex Chronic Patients Support Service (CCPSS). The service is staffed by nurse case managers and internal medicine physicians with the support of community-based social workers. Its activity is centered on nurse-led case management, the development of a shared individualized care plan, partnerships with community services to create solutions that optimize the management of patients’ chronic conditions, and close clinical involvement of internal medicine physicians. The intervention combines clinical, organizational, and professional integration mechanisms.

Follow-up is provided through regular home visits by nurses, complemented by telephone case management and by hospital- or home-based urgent or scheduled multidisciplinary consultations involving physicians and nurses. The primary objective of the intervention is to optimize the self-management of chronic conditions by patients and/or caregivers, combined with clinical surveillance that enables early detection of disease exacerbations, thereby reducing the number of emergency department visits and hospitalizations. Internal medicine was selected as the anchor specialty, with the aim of reducing the need for multiple hospital outpatient consultations.

CCPs may be referred to the team by any physician, regardless of the level of care. Referrals may originate from hospital inpatient wards, outpatient clinics, emergency departments or primary care consultations. The initial assessment is multidisciplinary and is conducted at the patient’s home, with the presence of caregivers whenever available. A comprehensive assessment is performed covering the physical, social, environmental, and mental domains, with particular emphasis on the problems most valued by the patient. Medication reconciliation is performed to identify discrepancies in the prescribed therapies. Based on the information gathered, an individualized care plan is collaboratively developed by the multidisciplinary team and discussed with patients and caregivers to promote shared decision-making regarding the proposed interventions and management strategies. The intervention is grounded in person-centered care principles, incorporating patient and caregiver priorities into the shared care planning process. This plan outlines both pharmacological and non-pharmacological measures aimed at improving chronic disease management, promoting self-management, and proactively addressing factors associated with acute decompensation and healthcare utilization. In addition, the shared care plan functions as a coordination tool across care settings, facilitating communication and continuity among healthcare professionals involved in the patient’s care pathway. Partnerships with existing community resources, including the municipal police, day care centers, and trained volunteers who support medication box preparation, are also explored.

Case management nurses follow a maximum of 30 patients concurrently, with the frequency of home visits determined according to each patient’s individual needs. Physicians work closely with case managers and can undertake both scheduled and urgent home visits as well as provide consultancy without the patient’s presence in situations where changes in the clinical strategy are required. Their responsibilities also include deprescribing in selected cases and simplifying medication regimens to enhance adherence.

The team liaises with both primary care and hospital-based teams through regular contact, enabling discussion of clinical strategies and sharing of information. Whenever possible, reducing the number of contacts with health services outside the team is encouraged to minimize the fragmentation of care. Home-based multidisciplinary follow-up enables continuity across care transitions. Patient follow-up by the team is delivered at different levels of intensity, with closer and more frequent contact for those recently enrolled and for patients who continue to experience difficulties managing their chronic conditions. Depending on the level of follow-up required, the team’s intervention may include face-to-face and/or phone-based case management, scheduled consultations, or consultancy support provided to the Family Health Team.

The intervention is not intended to replace existing healthcare services but to improve coordination between primary care, hospital care and community resources while reducing fragmentation of care.

### 2.4. Healthcare Utilization and Provider Cost Analysis

The CCPSS belongs to a Local Health Unit that functions under a single administrative structure integrating the hospital and primary care centers and is responsible for a geographically defined population of approximately 173,000 inhabitants. The Local Health Unit structure facilitates vertical integration between hospital and primary care services. It applies a departmental cost allocation system using homogeneous cost pools and the reciprocal method to account for interdepartmental support. Healthcare utilization included emergency department visits, inpatient admissions, inpatient bed-days, outpatient consultations, primary care consultations and day hospital activity. Provider costs were estimated using average unit costs for each healthcare activity; therefore, the reported cost savings represent average rather than marginal cost estimates. The unit cost of each healthcare service is presented in Table 1. The higher average cost of a day hospital attendance reflects the nature of the services provided; specifically, day hospital episodes include high-cost treatments, such as chemotherapy and biological therapies, together with the associated specialized personnel, facilities and overhead costs. In accordance with the contractualization of the Local Health Unit, the CCPSS unit of measurement is defined in equivalence to outpatient attendance, whereby open consultations, home visits, and consultancy activities are considered individual units.

Unit costs were derived from the average direct and indirect costs for 2024, as detailed in the cost components (see Supplementary Table S1). Total costs were calculated by multiplying the volumes of care with their corresponding unit prices and are presented in euros. From the healthcare provider perspective, the only costs not included were those related to medications dispensed through community pharmacies. Costs related to social care services, long-term care and informal caregiving were also outside the scope of this economic evaluation, as they fall beyond the healthcare provider perspective adopted in this study.

### 2.5. Statistical Analysis

Owing to the different follow-up durations, analyses were performed on subsets of patients with complete data at each time horizon. A three-time-point analysis was conducted in patients with at least one year of follow-up, whereas a four-time-point analysis, including two years after the intervention, was restricted to patients with complete two-year follow-up. Regarding the use of health services, repeated measures were compared using the *Friedman* test, followed by pairwise *Wilcoxon* signed-rank tests comparing the year prior to program integration with one and two years after integration. Statistical analyses were performed using IBM SPSS Statistics for Windows, version 28.0 (IBM Corp., Armonk, NY, USA).

Analyses at each follow-up time point were restricted to patients with complete follow-up data for that period. Patients who died, were referred to palliative care, moved residence or discontinued follow-up were excluded from subsequent longitudinal analyses. Baseline characteristics of patients completing and not completing follow-up were compared.

The endpoints for the cost-outcomes analysis were the reduction in emergency department visits and hospitalization days per patient. The mean healthcare cost reductions per avoided event indicator were calculated by dividing the mean reduction in total annual healthcare costs per patient by the corresponding mean reduction in healthcare utilization (emergency department visits and hospital bed-days). Uncertainty was assessed using non-parametric bootstrapping with 1000 replications at the patient level. The mean cost and effect differences were plotted on the cost-outcome plane.

## 3. Results

During the study period, 473 patients were enrolled in the CCPSS. A total of 308 and 237 patients completed one- and two-year follow-up, respectively. Of the 165 patients who did not complete one year of follow-up, 125 died, 15 were referred to the Palliative Care Team, 13 changed residence, and 12 did not benefit from the team’s intervention. An additional 71 patients did not complete the second year of follow-up. Among these, 56 died, eight were referred to the Palliative Care Team, four changed residence, and three did not derive benefit from the team’s intervention.

Table 2 summarizes the characteristics of the included patients. The study population represented a highly complex cohort of older adults with multimorbidity, functional impairment, polypharmacy and intensive use of healthcare services. Participants had a median age of 81.9 years, 56.7% were women, and they were receiving a mean of 10.6 chronic medications. During the two years and one-year preceding enrollment, respectively, patients attended a mean of 2.7 and 3.6 emergency department episodes, 0.7 and 1.4 hospital admissions accounting for 6.5 and 14.9 inpatient bed-days, 6.9 and 7.7 hospital outpatient consultations, and 11 and 12.8 primary care consultations. Day hospital utilization was comparatively infrequent (mean 0.2 and 0.3 sessions per patient). Heart failure (82.5%), diabetes (66.2%) and chronic kidney disease (59.4%) were the most prevalent chronic conditions.

Table 3 (Panels A and B) summarizes the changes in healthcare utilization before and after the intervention over one-year and two-year follow-up periods.

Across both analyses, emergency department visits, inpatient bed-days, outpatient attendances, and primary care consultations decreased following the intervention. In the one-year analysis (Table 3 Panel A), all outcomes showed statistically significant reductions compared with the pre-intervention period (all Friedman tests *p* < 0.001), with consistent results in pairwise comparisons.

These reductions were sustained over the long term (Table 3 Panel B), with lower healthcare utilization persisting for up to 2 years after the intervention across all outcomes examined (all Friedman tests *p* < 0.001).

Table 4 (Panels A and B) presents the total healthcare costs before and after the intervention over one-year and two-year follow-up periods, respectively.

In both analyses, the total mean cost per patient was lower following the intervention. In the one-year analysis (Table 4 Panel A), the mean post-intervention costs decreased compared with those in the pre-intervention period. This reduction was sustained over the long term (Table 4 Panel B), with mean costs remaining lower for up to two years after the intervention. Overall, these findings demonstrate a reduction in healthcare costs after implementing the intervention.

**Figure 1:**
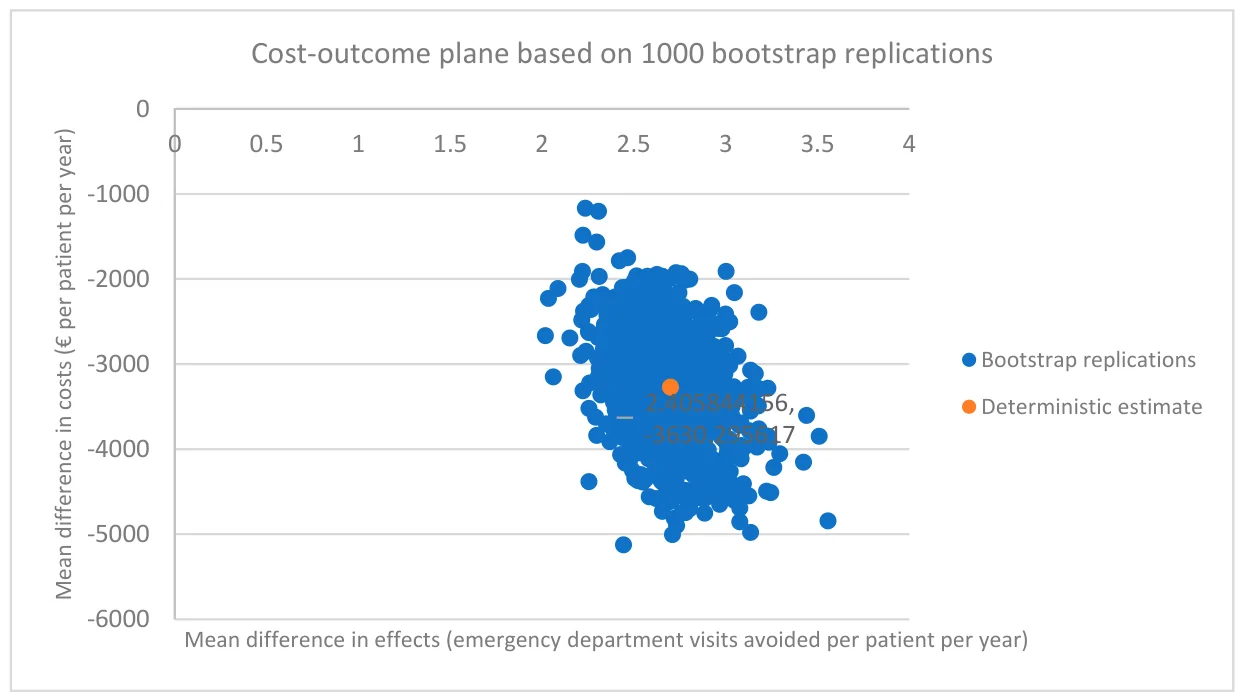
Cost-outcome plane for emergency department visits avoided per patient per year. Most bootstrap replications were located in the southeast quadrant, suggesting lower costs and fewer emergency department visits associated with the intervention.

**Figure 2:**
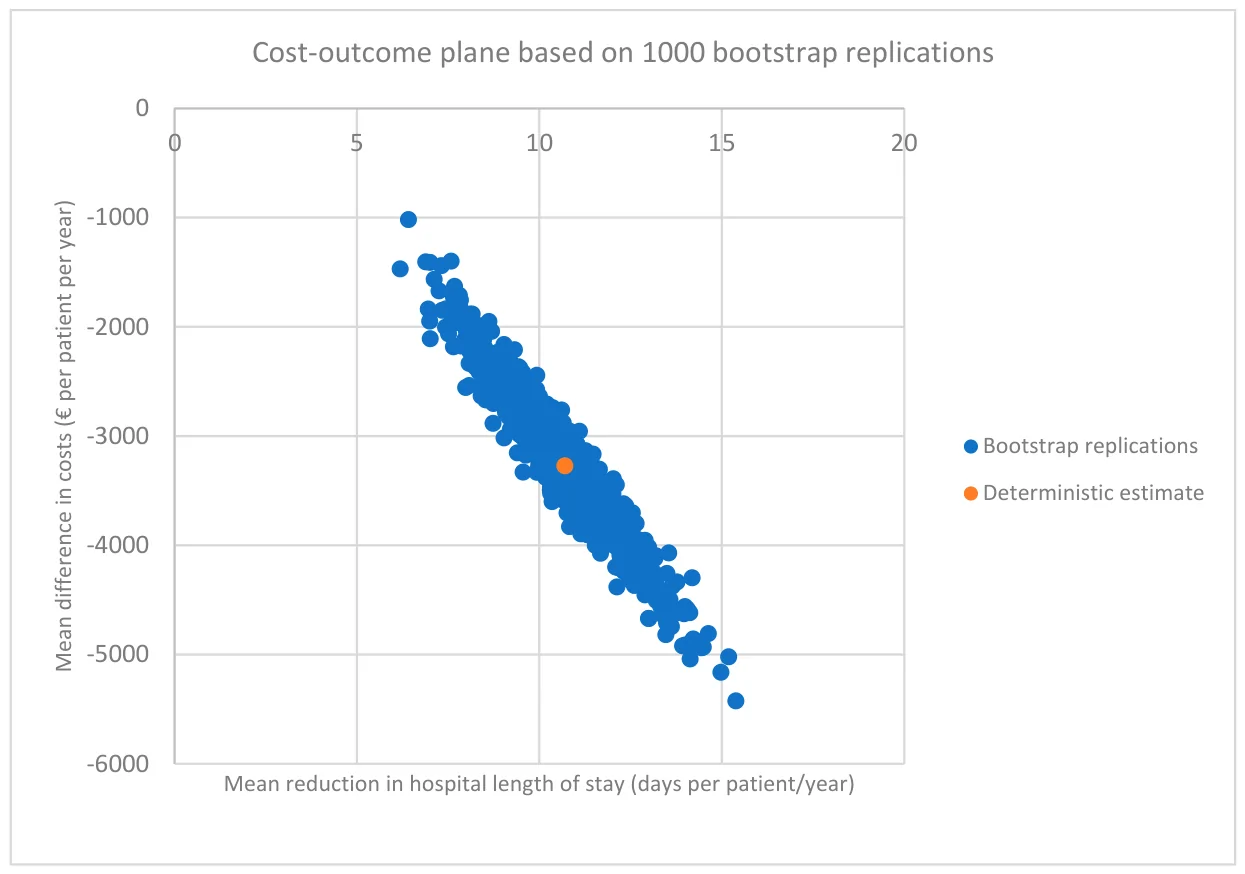
Cost-outcome plane for mean reduction in hospital length of stay (days per patient/year). Most bootstrap replications were located in the southeast quadrant, suggesting lower costs and reduced hospital length of stay associated with the intervention.

Table 5 (Panels A and B) summarizes the results of the exploratory economic analyses. In Panel A, emergency department visits and days of hospitalization were analyzed as separate outcomes in patients who completed one year of follow-up. Panel B presents the same analyses for patients who completed two years of follow-up. Across both follow-up periods, lower healthcare costs occurred alongside reductions in acute healthcare utilization. These findings were further supported by the sensitivity analysis. The cost-outcome planes based on 1000 bootstrap replications for emergency department visits at one year (Figure 1) and for days of hospitalization over the same period (Figure 2) located the majority of replications within the southeast quadrant, indicating lower provider costs associated with lower acute healthcare utilization.

## 4. Discussion

This study evaluated the implementation of a multidisciplinary integrated care program for CCPs within routine clinical practice. The program was associated with sustained reductions in emergency department visits, inpatient bed-days and provider costs over two years of follow-up. These findings suggest that integrated longitudinal care may contribute to more coordinated healthcare delivery for patients with complex chronic conditions.

Reductions in emergency department visits, inpatient bed-days, outpatient attendances, and primary care consultations were observed following program implementation and remained sustained over the long-term follow-up. The magnitude and persistence of these changes suggest that the program effectively addressed the key drivers of unplanned healthcare utilization commonly observed among patients with multimorbidity, recurrent exacerbations, and fragmented care pathways. The sustained effects over two years indicate that the observed benefits were not limited to short-term clinical stabilization but may reflect improvements in care coordination, continuity of care, proactive clinical monitoring, and anticipatory management strategies. Therefore, the reduction in acute healthcare utilization may reflect not only improved clinical stability but also enhanced integration between hospital and community-based care, with reduced fragmentation across care settings. The intervention promoted continuity through regular home follow-up, shared care planning, proactive surveillance and close collaboration between hospital and primary care teams.

The CCPSS intervention led to an increase in the number of home-based and outpatient consultations (when the team’s interventions were added to those provided by primary care services). There was a redistribution of healthcare utilization, namely emergency department visits and hospital admissions, by sustained and close follow-up delivered by healthcare teams, a finding that has been previously described [15].

From the provider perspective, the program was associated with lower overall healthcare costs, largely driven by reductions in emergency department use and inpatient care. These findings are particularly relevant, as inpatient and emergency services represent the largest contributors to healthcare expenditures among complex patients. However, in publicly funded hospital systems, a substantial proportion of healthcare costs are fixed in the short term [16]. Consequently, hospital bed-days freed through reduced utilization are frequently reallocated to other patients rather than translating into immediate lower provider costs [17]. Consequently, the marginal cost savings associated with reduced hospital activity are often substantially lower than the corresponding average costs that are typically used in economic evaluations. Nevertheless, increasing evidence indicates that case management programs and other interventions that enhance ambulatory approaches to chronic disease management may help to promote a shift in healthcare delivery models, favoring more home-based care and greater flexibility, with potential implications for reducing the fixed costs associated with highly structured hospital-based systems that are less responsive to changes in demand [18]. The reallocation of hospital resources towards these programs has the potential to scale up benefits and lower provider costs.

Exploratory economic analyses consistently showed that lower provider costs occurred alongside reductions in acute healthcare utilization, supporting the internal consistency of the observed findings. The exploratory economic analysis after two years suggests that, over time, the intervention becomes less costly, with a lower mean cost in the second year relative to the first, while maintaining the observed reductions in healthcare utilization. This pattern, as reported previously in the literature [19], may reflect greater clinical stabilization of the patients under follow-up and more efficient use of resources, underscoring the importance of economic evaluations with longer time horizons.

These results align with previous evidence indicating that integrated, multidisciplinary care models can reduce acute healthcare utilization among patients with complex needs [20]. However, many of these interventions have reported mixed economic findings or only short-term effects [21]. Our findings extend previous evidence showing that integrated multidisciplinary care can reduce fragmentation and acute healthcare utilization among patients with multimorbidity. Unlike many disease-specific interventions [22], the present program targeted patients with heterogeneous chronic conditions through a person-centered integrated care approach embedded within routine clinical practice. Although implemented within the Portuguese National Health Service, the intervention addresses challenges common to many health systems, namely fragmentation of care, rising multimorbidity, and pressure on acute hospital services.

These findings have practical implications for healthcare organizations facing increasing demand from aging populations with multimorbidity. Rather than increasing the volume of healthcare delivered, integrated multidisciplinary programs may contribute to reorganizing healthcare delivery towards more proactive, coordinated and person-centered care.

Several limitations should be considered when interpreting these findings. The before-and-after study design does not allow causal attribution with the same confidence as a randomized controlled trial. Residual confounding arising from changes in patients’ clinical trajectories or healthcare organization over time cannot be excluded. Secular trends or regression to the mean may have also influenced outcomes. Nevertheless, the consistency of findings across multiple healthcare utilization outcomes and two follow-up periods strengthens confidence in the observed associations.

Selection bias is an important consideration in this study. Patients were not randomly selected but referred to the integrated care program based on clinical judgment and predefined criteria for complexity. As a result, the study population may represent a subset of CCPs who were considered both at high risk and potentially responsive to structured care. Therefore, results should not be interpreted as representative of all CCPs but rather of those engaged in thorough integrated care.

The study population was characterized by high morbidity and mortality, reflecting the clinical reality of patients referred to complex care services. While this enhances external validity for similar populations, mortality and transitions to palliative care may have influenced utilization patterns over time, as patients with poorer prognoses may have had shorter follow-up periods due to death. To ensure comparable pre- and post-enrollment observation periods, healthcare utilization and cost analyses were restricted to patients who completed the predefined one- and two-year follow-up intervals. Including patients with incomplete follow-up would have resulted in unequal observation windows, potentially introducing bias into comparisons of healthcare utilization and provider costs. However, such transitions are intrinsic to complex care populations and were considered part of the program’s intended scope rather than unintended attrition. To address the survival bias, we conducted separate analyses at 12 and 24 months, comparing baseline characteristics of survivors and non-survivors at each time point (see Supplementary Table S2). Patients who failed to complete follow-up were older and showed lower functional status. These findings suggest a non-random attrition process driven mainly by baseline frailty. Nonetheless, the absence of uniformly higher baseline healthcare utilization among non-survivors suggests that improvements observed among survivors are not solely explained by attrition of the most resource-intensive patients.

Another limitation was the exclusion of costs outside the healthcare provider perspective. Community pharmacy costs, as well as costs related to social care services, long-term care and informal caregiving, were not included in the economic evaluation. According to unpublished data, the mean number of prescribed medications increased from 10.6 to 11.5 per patient one year after enrollment in the program, an increase that is unlikely to have had a substantial impact on overall costs [23]. However, although reductions in healthcare utilization and provider costs were observed, the possibility that some costs were shifted to social care or other non-healthcare sectors cannot be excluded.

The use of average costs for the health services provided may underestimate the true costs associated with CCPs, whose clinical complexity typically entails higher expenditure on diagnostic tests and medications, potentially reducing the observed magnitude of the financial impact associated with reduced healthcare utilization.

Despite these limitations, the study offers important implications for healthcare systems facing increasing demand from patients with complex needs. The findings suggest that well-structured complex care programs are associated with improved healthcare utilization outcomes and lower provider costs, supporting their role as high-value interventions within constrained healthcare budgets. Beyond the observed reductions in healthcare utilization and provider costs, integrated multidisciplinary care programs may also benefit family caregivers by improving care coordination, facilitating access to healthcare services and reducing the need for unplanned hospital use. Although caregiver burden was not directly assessed in this study, these organizational improvements may contribute to a more sustainable caregiving experience for families supporting patients with complex chronic conditions.

Overall, this study supports integrated multidisciplinary care as a feasible strategy to improve healthcare delivery for CCPs while reducing reliance on acute hospital-based services and lowering provider costs within routine clinical practice. Future research should evaluate these programs using comparative designs, adopt broader societal perspectives and incorporate patient-reported outcomes to better characterize their overall impact.

## 5. Conclusions

This real-world evaluation showed that the implementation of a multidisciplinary integrated care program for CCPs was associated with sustained reductions in emergency department utilization, inpatient bed-days and overall provider costs over two years of follow-up. These findings suggest that integrated multidisciplinary care may improve the organization and coordination of healthcare delivery for patients with multimorbidity while reducing reliance on acute hospital-based services. Although causal inference cannot be established, the consistency of the findings across multiple healthcare utilization outcomes supports further implementation and prospective evaluation of similar integrated care programs.

## Figures and Tables

**Table 1:** Unitary cost of each health service provided.

Cost Variable	Unitary Cost (Euros)
Primary care (general practice) consultation	108.25
Outpatient attendance	201.65
Medical inpatient bed-day	384.91
Surgical inpatient bed-day	556.20
Emergency department episode	211.76
Day hospital session	426.94

**Table 2:** Characteristics of patients at baseline (*n* = 473).

Characteristic	Value
Age in years, median (IQR)	81.9 (77.1–86.1)
Female sex, *n* (%)	268 (56.7%)
Number of drugs, mean (SD)	10.6 (3)
Barthel Index (SD)	70.6 (30.6)
Emergency episodes in the last 24 months, mean (SD)	2.7 (4.3)
Emergency episodes in the last 12 months, mean (SD)	3.6 (4)
Number of hospitalizations in the last 24 months, mean (SD)	0.7 (1)
Number of hospitalizations in the last 12 months, mean (SD)	1.4 (1)
Days of hospitalization in the last 24 months, mean (SD)	6.5 (13.2)
Days of hospitalization in the last 12 months, mean (SD)	14.9 (20.7)
Number of hospital consultations in the last 24 months, mean (SD)	6.9 (7)
Number of hospital consultations in the last 12 months, mean (SD)	7.7 (7)
Number of primary care consultations in the last 24 months, mean (SD)	11 (7.6)
Number of primary care consultations in the last 12 months, mean (SD)	12.8 (8.3)
Number of day hospital sessions in the last 24 months, mean (SD)	0.2 (1.2)
Number of day hospital sessions in the last 12 months, mean (SD)	0.3 (1.7)
Chronic diseases, mean (SD)	3.2 (0.8)
Chronic conditions, *n* (%)	
-Chronic respiratory failure	245 (51.8%)
-Chronic kidney disease	281 (59.4%)
-Heart failure	390 (82.5%)
-Active cancer	48 (10.1%)
-Cerebrovascular disease	232 (49.0%)
-Diabetes	313 (66.2%)
-Chronic liver disease	25 (5.3%)

IQR—Interquartile range; SD—Standard deviation.

**Table 3:** Healthcare utilization before and after the intervention.

Panel A—One-Year Follow-Up (*n* = 308)											
Outcomes	Two Years Before Median (IQR)		One Year Before Median (IQR)		One Year After Median (IQR)		Friedman χ^2^		*p* Value	Wilcoxon Z (−1 vs. +1)	*p* Value
**Emergency department episodes**	2 (4)		3 (4)		1 (2)		160.98		<0.001	−11.38	<0.001
**Inpatient bed-days**	0 (8)		10 (25)		0 (7)		98.72		<0.001	−7.91	<0.001
**Outpatient attendances**	5 (9)		6 (8)		5 (7)		13.591		<0.001	−3.03	<0.002
**Primary care consultations**	9 (9)		11 (9)		9 (9)		38.452		<0.001	−7.230	<0.001
**Panel B** **—** **Two** **-** **Year Follow** **-** **Up (*n* = 237)**											
**Outcomes**	**Two Years Before Median (IQR)**	**One Year Before Median (IQR)**		**One Year After Median (IQR)**		**Two Years After Median (IQR)**		**Friedman χ^2^**	***p* Value**	**Wilcoxon Z (−1 vs. +2)**	***p* Value**
**Emergency department episodes**	1 (4)	3 (5)		1 (2)		1 (2)		180.957	<0.001	−10.911	<0.001
**Inpatient bed-days**	0 (7)	9 (24)		0 (5)		0 (3)		122.406	<0.001	−8.402	<0.001
**Outpatient attendances**	5 (9)	6 (8)		5 (6)		3 (5)		79.716	<0.001	−7.744	<0.001
**Primary care consultations**	9 (8)	11 (9)		9 (9)		8 (9)		44.531	<0.001	−6.504	<0.001
**CCPSS consultations**	NA	NA		7 (10)		6 (8)		NA	NA	(+1 vs. +2) −4.59 ^a^	<0.001

NA—not applicable. IQR—Interquartile range. CCPSS—Complex Chronic Patients Support Service. ^a^ Wilcoxon signed-rank test comparing CCPSS consultations between one-year and two-year follow-up consultations.

**Table 4:** Annual healthcare costs per patient before and after the intervention.

Panel A—One-Year Follow-Up (*n* = 308)					
Cost Category	Two Years Before (Euros)	One Year Before (Euros)		One Year After (Euros)	
**Emergency department episodes**	562	933		363	
**Medical inpatient bed-days**	2031	6273		2272	
**Surgical inpatient bed-days**	594	716		598	
**Outpatient attendances**	1411	1578		1389	
**Primary care consultations**	1143	1346		1033	
**Day hospital sessions**	74	135		135	
**CCPSS consultations**				1977	
**Total healthcare cost per patient**	5816	10,980		7753	
**Panel B—Two-Year Follow-Up (*n* = 237)**					
**Cost Category**	**Two Years Before (Euros)**	**One Year Before (Euros)**	**One Year After (Euros)**		**Two Years After (Euros)**
**Emergency department episodes**	521	874	328		290
**Medical inpatient bed-days**	2002	6150	1773		1224
**Surgical inpatient bed-days**	580	683	570		467
**Outpatient attendances**	1457	1628	1326		958
**Primary care consultations**	1125	1334	972		994
**Day hospital sessions**	56	148	117		94
**CCPSS consultations**			1957		1602
**Total healthcare cost per patient**	5743	10,817	7130		5630

CCPSS—Complex Chronic Patients Support Service.

**Table 5:** Exploratory cost-outcome analysis of the intervention.

Panel A—One-Year Follow-Up (*n* = 308)			
Healthcare Utilization and Cost-Outcomes	One Year Before	One Year After	Increment
**Mean annual healthcare cost per patient (Euros)**	10,980	7753	−3227
**Mean emergency department visits per patient**	4.4	1.71	−2.69
**Mean inpatient bed-days**	16.4	7	−9.4
**Mean healthcare cost reduction per avoided emergency department visit (Euros)**			−1200 (cost saving)
**Mean healthcare cost reduction per avoided inpatient bed-days (Euros)**			−343 (cost saving)
**Panel B—Two Year Follow Up (*n* = 237)**			
**Healthcare Utilization and Cost-Outcomes**	**One Year Before**	**Two Years After**	**Increment**
**Mean annual healthcare cost per patient (Euros)**	10,817	5630	−5187
**Mean emergency department visits per patient**	4.13	1.37	−2.76
**Mean inpatient bed-days**	17.2	4	−13.2
**Mean healthcare cost reduction per avoided emergency department visit (Euros)**			−1879 (cost saving)
**Mean healthcare cost reduction per avoided inpatient bed-days (Euros)**			−393 (cost saving)

## Data Availability

The datasets used in the current study are available from the corresponding author upon reasonable request.

## Supplementary Materials

The following supporting information can be downloaded at: https://www.mdpi.com/article/10.3390/healthcare14162542/s1, Table S1: Cost components included in direct and indirect costs; Table S2: Baseline characteristics of survivors and non-survivors at one- and two-year landmark time points.

## Abbreviations

The following abbreviations are used in this manuscript:

CCP	Complex chronic patient
CCPSS	Complex Chronic Patients Support Service
IQR	Interquartile range
SD	Standard deviation
