# Supplementary material for: A Multidisciplinary Integrated Care Program for Complex Chronic Patients: A Real-World Evaluation of Healthcare Utilization and Provider Costs
Source: Healthcare (Basel). 2026 Aug 14;14(16):2542. doi: 10.3390/healthcare14162542 (PMC13511912; doi:10.3390/healthcare14162542)
Supplement: Supplementary file 1 [file healthcare-14-02542-s001.zip › healthcare-4437862-supplementary.pdf]

Supplementary Material
Supplementary Table S1: cost components included in direct and indirect costs.

DIRECT COSTS	INDIRECT COSTS
**Goods**	**Diagnostic and Therapeutic Services**
Consumable materials<br>Pharmaceutical products<br>Clinical consumables<br>Food products<br>Hotel and catering consumables<br>Administrative consumables<br>Maintenance and repair materials<br>Other consumable materials	Cytopathology<br>Histopathology<br>Autopsies<br>Biochemistry<br>Haematology<br>Immunology<br>Microbiology<br>Radiology<br>Angiography<br>Mammography<br>Computed tomography (CT)<br>Ultrasound<br>Magnetic resonance imaging (MRI)<br>Physical and rehabilitation medicine<br>Immunohaematology and transfusion medicine
**External Supplies and Services**<br>Subcontracted services<br>External supplies and services	**Specialist Diagnostic Techniques**<br>Cardiology investigations<br>Gastroenterology investigations<br>Neurology investigations<br>Ophthalmology investigations<br>Ear, nose and throat (ENT) investigations<br>Respiratory medicine investigations<br>Urology investigations<br>Gynaecology investigations<br>Dermatology investigations<br>Nephrology investigations<br>Obstetrics investigations
**Current Transfers Granted – Social Benefits**	**Clinical Support Services**<br>Anaesthesiology<br>Chronic pain management<br>Operating theatre<br>Nutrition<br>Sterilisation services<br>Psychology<br>Social work<br>Pharmacy<br>In-hospital palliative care team<br>Hospital infection prevention and control service
**Staff Costs**<br>Remuneration of Governing Bodies<br>**Staff Remuneration**<br>Basic staff remuneration<br>Senior management staff<br>Medical staff<br>Nursing staff<br>Diagnostic and therapeutic technical staff<br>Senior technical staff<br>Administrative staff<br>Operational support staff<br>Information technology staff<br>Other staff	**Hotel, Logistics and Facilities Services**<br>Catering and dietetics service<br>Laundry service<br>Cleaning and hygiene service<br>Security and support services<br>Barber services<br>Grounds and gardens maintenance<br>Transport services<br>Waste collection and disposal service<br>Mortuary services<br>**Management and Administrative Services**<br>Hospital administration and executive management<br>Financial management department

Additional Pay and Allowances

Overtime work
Overtime hours
On-call duties
Shift work
Night duty and allowances
Shift allowance
Cash-handling allowance
Meal allowance
Travel and subsistence allowances
Uniforms and personal protective items
Food and accommodation
Other allowances

Direct Social Benefits

Holiday and Christmas bonuses
Performance-related bonuses
Pensions

Employer Social Contributions

Insurance for occupational accidents and diseases
Voluntary social contributions
Other staff-related costs

Procurement and logistics service
Human resources and records management department
Patient admissions service
Information technology service
Education and research
Legal department
Patient liaison service
Clinical coding department
Quality management
Risk management coordination office
Internal audit
Freedom of information officer
Contracting and commissioning office
Communications and public relations office
Telephone exchange
Primary Care Cluster management support unit
Discharge management team
Occupational health service
Nursing supervision
Technical committees
Haemodialysis Clinic protocol
Spiritual care service
Records management
Archive
Visiting services
Planning and performance management service
Training centre
Outpatient department support
Clinical trials center
Hospital transfusion committee unit

Supplementary Table S2: Baseline characteristics of survivors and non-survivors at one- and two-year landmark time points.

	Not completed one year (n = 165)	One Year follow-up (n=308)	Not completed two years (n=71)	Two Years follow-up (n=237)
Age	82.8	80	80.4	80
Female Sex	55.8%	57,1%	61.2%	55.7%
Barthel index	60	76	70	78
Emergency Episodes, mean	2	4.4	5.3	4.1
Days of hospitalization, mean	9.8	17.7	19.3	17.2
Number of hospital consultations, mean	7.5	7.8	7	8
Number of primary care consultations, mean	13.3	12.5	13	12.3
Chronic diseases, mean (SD	3.2	3.2	3.2	3.3
Chronic respiratory failure	47.9%	53.9%	56.3%	53.2%
Chronic kidney disease	58.8%	59,7%	64.5%	58.2%
Heart failure	84.8%	81.2%	77.5%	82.3%
Active cancer	12.1%	9%	7%	9.7%
Cerebrovascular disease	53.3%	46.7%	39.4%	48.9%
Diabetes	61.2%	68.8%	63.4%	70.5%
Chronic liver disease	3.6%	6.2%	8.4%	5.5%
Number of drugs, mean (SD)	10.7	10.5	10.8	10.5
